# Selective ion sensing with high resolution large area graphene field effect transistor arrays

**DOI:** 10.1038/s41467-020-16979-y

**Published:** 2020-06-26

**Authors:** Ibrahim Fakih, Oliver Durnan, Farzaneh Mahvash, Ilargi Napal, Alba Centeno, Amaia Zurutuza, Viviane Yargeau, Thomas Szkopek

**Affiliations:** 1 Department of Electrical and Computer Engineering, McGill University, Montreal, QC H3A 2A7 Canada; 2 Graphenea Semiconductor S.L.U, Paseo Mikeletegi 83, 20009 San Sebastian, Spain; 3 Department of Chemical Engineering, McGill University, Montreal, QC H3A 0C5 Canada

**Keywords:** Sensors, Electrical and electronic engineering, Electronic properties and devices, Design, synthesis and processing

## Abstract

Real-time, high resolution, simultaneous measurement of multiple ionic species is challenging with existing chromatographic, spectrophotometric and potentiometric techniques. Potentiometric ion sensors exhibit limitations in both resolution and selectivity. Herein, we develop wafer scale graphene transistor technology for overcoming these limitations. Large area graphene is an ideal material for high resolution ion sensitive field effect transistors (ISFETs), while simultaneously enabling facile fabrication as compared to conventional semiconductors. We develop the ISFETs into an array and apply Nikolskii–Eisenman analysis to account for cross-sensitivity and thereby achieve high selectivity. We experimentally demonstrate real-time, simultaneous concentration measurement of K^+^, Na^+^, ${{\rm{NH}}}_{4}^{+}$, ${{\rm{NO}}}_{3}^{-}$, ${{\rm{SO}}}_{4}^{2-}$, ${{\rm{HPO}}}_{4}^{2-}$ and Cl^−^ with a resolution of $\sim\! 2\times 1{0}^{-3}\,{\mathrm{log}}\,$ concentration units. The array achieves an accuracy of  ±0.05 log concentration. Finally, we demonstrate real-time ion concentration measurement in an aquarium with *lemnoideae lemna* over three weeks, where mineral uptake by aquatic organisms can be observed during their growth.

## Abstract (web summary)

The authors demonstrate wafer-scale, graphene-based ion sensitive field effect transistors arrays for simultaneous concentration measurement of K^+^, Na^+^, NH_4_^+^, NO_3_^−^, SO_4_^2−^, HPO_4_^2−^ and Cl^−^, and use their technology for real-time ion concentration measurements in an aquarium with lemnoideae lemna over a period of three weeks.

## Introduction

Measuring the ion content of analytes at low concentration, accurately, quickly, and reliably is important in a diverse range of applications, including genome sequencing^1^, medical diagnostics^2^, environmental monitoring^3,4^, and industrial process control^5^. Potentiometric ion sensors, especially solid-state sensors, have been attracting increasing interest as compared to state-of-the-art chromatographic^6^ and spectrophotometric^7^ techniques. Potentiometric sensors are compact, easy to integrate with electronics, and have the capacity for real-time, on-site measurements. The most common solid-state ion sensors are the ion sensitive field effect transistors (ISFETs), where the gate of the ISFET incorporates an ion selective membrane in the form of either a glass or ionophore mixture^8^. The principle of operation relies on Nernst’s law. Ions reversibly bind to the membrane, which acts as a buffer in a well-designed ISFET, thus creating an electric potential in proportion to the logarithm of ion concentration. The surface potential and transistor channel current of the ISFET is thus modulated by ion concentration. However, the selectivity of ion selective membranes is not absolute. Ions other than the target ion can also reversibly bind to the membrane, leading to an interfering response in potentiometric sensors that typically leads to unreliable measurement of ion concentration in an analyte with multiple ionic species. This can be overcome by assembling ISFETs for a variety of ionic species, including known interfering ions, and subsequently applying Nikolskii–Eisenman analysis to estimate ion concentration^9–11^. While the concept of ion sensitive arrays has been demonstrated in the past to overcome poor selectivity^12–14^, these arrays have faced challenges in achieving the resolution and detection limits required for many real-time sensing applications.

Current state-of-the-art ISFETs are silicon-based and fabricated using standard CMOS processes, where the ion selective membrane is added through back end of line processing. Silicon ISFETs are typically micro-scaled, and arrays of up to 10^7^ ISFETs have been integrated for measuring pH^1,15,16^. However, as the active area of ISFETs is scaled down, the effects of low-frequency charge fluctuation become more pronounced, and it becomes more difficult to measure ion concentration with high resolution^17^. It has been previously shown that in order to improve resolution in ion concentration measurement, it is important to maximize channel area, charge carrier mobility, and capacitance between the ISFET channel and ion binding sites^18^. Consequently, it is impractical to fabricate large-area silicon ISFET arrays to account for ion interference with multiple ISFETs while simultaneously achieving reliable and high resolution sensor response. Efforts to improve resolution have been directed in recent years to the study of alternative materials and structures, including Si nanowires^19–21^, combined silicon FET and bipolar junction transistors^22^, AlGaN/GaN high electron mobility transistors (HEMTs)^23,24^, exfoliated MoS_2_^25,26^, InN grown by molecular beam epitaxy^27^ and carbon nanotubes^28^ as summarized in Table 1. All sensor resolutions are reported in Table 1 with a 90% confidence level in accord with IUPAC guidelines^29^.

**Table 1:** Comparison of recent ISFETs in literature.

Channel material	Target ion	Sensitivity (mV per decade)	Resolution (log concentration (M)).
Si^1,15,16^	H^+^	58^1^, 41^15^, 46^16^	0.02^1^, 0.08^16^
Si nanowires^19–21^	H^+^	48^19^, 40^21^	0.05^21^
Si FET + BJT^22^	H^+^	20^22^	0.005^22^
AlGaN/GaN^23,24^	${{\rm{NH}}}_{4}^{+}$	55^23^	>0.2^24^
MoS_2_^25^	H^+^	58.7	0.02
InN^27^	H^+^	58.3	0.03
Carbon nanotube^28^	K^+^	–	>0.06
Graphene^18,34^	H^+^, K^+^	55^18^, 37^34^	0.0003^18^, 0.002^34^

On the other hand, graphene ISFETs are optimal for overcoming these challenges because  ~cm^2^ large-area devices with charge carrier mobilities reaching up to 7000 cm^2^ V^−1^ s^−1^ can be fabricated using facile chemical vapor deposition methods and thin film deposition techniques^18^. Due to challenges involved in functionalizing graphene with sensing layers^30^, initial work on graphene ISFETs reported modest performance in terms of sensitivity^31^, range of detection^32^, and resolution^33^. We have previously shown that both metal oxides and ionophore selective membranes can be integrated with graphene ISFETs for H^+^ and K^+^ concentration measurement, respectively, operating at both thermodynamic and quantum limits with record detection limits and resolutions for potentiometric sensing, and excellent stability over the course of 5 months^18,34^.

In this paper, we report the demonstration of real-time, high resolution measurement of multiple ion concentrations using large-area graphene ISFETs that overcomes the challenge of ion interference. With wafer-scale processing, we fabricated large area graphene ISFETs (~cm^2^) with ionophore membranes as sensing layers to measure the concentration of K^+^, Na^+^, ${{\rm{NH}}}_{4}^{+}$, ${{\rm{NO}}}_{3}^{-}$, ${{\rm{SO}}}_{4}^{2-}$, ${{\rm{HPO}}}_{4}^{2-}$ and Cl^−^ down to concentrations lower than 10^−5^ M and a resolution of $\sim \!2\times 1{0}^{-3}\,{\mathrm{log}}\,$ concentration units. These ions were selected due to their prominence in agricultural runoff, and the resultant need to measure these ions for water quality monitoring^35,36^. We used the separate solution method to calibrate the elements of the ISFET sensor array and extract the Nikolskii selectivity coefficients of each sensor element to each ionic species in our study. With all sensor elements simultaneously measuring the same analyte solution, we used Nikolskii–Eisenman analysis to account for ion interference and accurately estimate multiple ion concentrations. By this method, we achieved an accuracy of  ±0.01 and  ±0.05 log concentration for cations and anions respectively. As a demonstration of the graphene ISFET array in operation, we monitored the uptake of ions by duckweed, *lemnoideae lemna*, in an aquarium over a period of three weeks. The graphene ISFET array performance is suitable for applications such as real-time water quality monitoring of ions of environmental concern^3^.

## Results

### Device structure and characterization

**Fig. 1:**
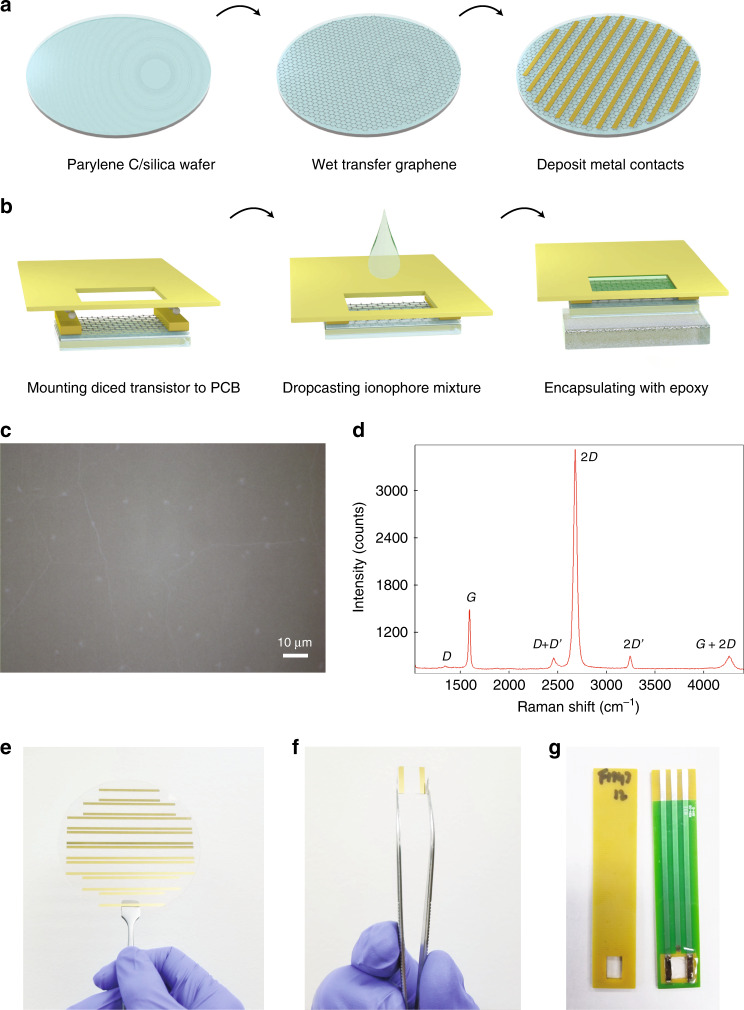
**Graphene ISFET Fabrication**. **a** Schematic of the fabrication process of the graphene ISFETs. 115 nm of parylene C was grown on a 100 mm diameter fused silica wafer. Graphene was grown via CVD on a 100 mm diameter Cu foil and wet-transferred to the target wafer using a PMMA handle. Ti/Au contacts were evaporated with the aid of a shadow mask. **b** The wafer was then diced into individual 1.1 cm × 1.1 cm devices, which were then mounted onto a PCB using silver epoxy. The ionophore mixture was dropcasted into the opening. Epoxy was used to encapsulate the back gate of the transistor. c Optical image with ×100  magnification of graphene monolayer transferred onto parylene C/fused silca wafer. Scale bar is 10 μm. **d** Raman spectra of 100 mm graphene wet-transferred onto target substrate. e An optical image showing a 4" graphene wafer on fused silica and parylene with gold contacts, ready to be diced into individual devices. **f** A single graphene device after being diced, and ready to be mounted on a PCB. fabrication process. **g** Top and bottom view of graphene ISFETs mounted on PCBs.

Graphene ISFETs were fabricated via wafer-scale processing methods, schematically illustrated in Fig. 1. A 100 mm diameter graphene monolayer grown via chemical vapor deposition (CVD) was wet-transferred onto a 100 mm target wafer. The wafer was 500 μm thick fused silica with a 115 nm layer of parylene C, a hydrophobic polymer that minimizes the unintentional doping of graphene and imparts stability^37^. A representative optical image of the transferred graphene with a ×100  magnification is shown in Fig. 1c. The quality of the transferred graphene was further confirmed via Raman spectroscopy, with a representative spectrum taken with a 532 nm pump wavelength shown in Fig. 1d along with peak assignments^38,39^. The D, G, and 2D peaks are located at 1341 ± 2 cm^−1^, 1593 ± 2 cm^−1^, and 2687 ± 2 cm^−1^, respectively. The D/G Raman intensity ratio is 0.045 ± 0.005 and the G/2D intensity ratio 0.027 ± 0.01, consistent with Raman spectra of monolayer graphene with a low density of defects and grain boundaries^38^. No particular effort was made at single-crystal graphene growth here, as grain boundaries in polycrystalline graphene have been shown to have a modest effect on graphene sheet resistance^40^.

After transferring the graphene onto the substrate, Ti/Au (20 nm/80 nm) contacts were evaporated onto the wafer to form source and drain contacts with the aid of a shadow mask. The wafer was then diced into individual 1.1 × 1.1 cm^2^ devices, and the devices were then mounted on a printed circuit board (PCB) with two part silver epoxy to contact the source and drain of each device (Fig. 1b). The PCBs have a 0.8 × 0.5 cm^2^ opening for the graphene FET to be exposed to the analyte solution. The 100 mm wafer was used to produce 52 graphene FETs, and the average two point resistance of the FET in air without an electrolytic gate was 277.0 Ω with a standard deviation of 11.9 Ω, indicative of the uniformity of wafer scale graphene FET processing.

To selectively measure the concentration of different ionic species in liquid, we used ionophore membranes for the following ions: K^+^, Na^+^, ${{\rm{NH}}}_{4}^{+}$, ${{\rm{NO}}}_{3}^{-}$, ${{\rm{SO}}}_{4}^{2-}$, ${{\rm{HPO}}}_{4}^{2-}$ and Cl^−^. These target ions were selected due to their prevalence in agricultural runoff^35,36^. The membranes were formed by drop-casting 50 μL of pre-prepared mixtures (see “Methods” section) onto the graphene through the PCB opening and left to dry overnight in ambient conditions. The membrane creates a seal between the graphene surface and PCB. Two component epoxy was applied and left to cure overnight to encapsulate the back of transistor and prevent electrical contact with the electrolytic solution. Figs. 1e–g show images image of the graphene ISFETs at different stages of the fabrication process.

To understand the evolution of the graphene monolayer through the ISFET fabrication process, electrical measurements were performed on graphene before and after the ionophore drop-casting. Back-gated field effect measurements were conducted on graphene ISFETs with similar substrates consisting of n-doped Si with 300 nm of dry thermal SiO_2_ and 115 nm of parylene C. Before the ionophore membranes were deposited, Hall mobilities up to 2050 cm^2^ V^−1^ s^−1^ were observed, and field effect mobilities up to  ~2100  cm^2^ V^−1^ s^−1^ were observed (see Supplementary Table 1). The ionophore membrane electron dopes the graphene monolayer, with anion ionophore membranes giving higher electron doping than cation membranes as seen in representative measurements for K^+^ and Cl^−^ ISFETs shown in Supplementary Fig. 1. The graphene FET carrier mobility does not change significantly during the ionophore deposition process, indicating that the ionophore membrane non-covalently functionalizes the graphene, preserving mobility while imparting ion sensitivity.

Additionally, measurements of Hall mobility and carrier density were made to study the uniformity of the graphene ISFETs from device to device. Based on a sampling of nine devices with active areas ranging from 25 μm^2^ to 26 mm^2^, the mean hole density was determined to be 8.27 × 10^12^ cm^−2^ with a standard deviation of 8.5 × 10^11^ cm^−2^. Likewise, the mean and standard deviation of the Hall mobility were 1720 cm^2^ V^−1^ s^−1^ and 240 cm^2^ V^−1^ s^−1^, respectively.

**Fig. 2:**
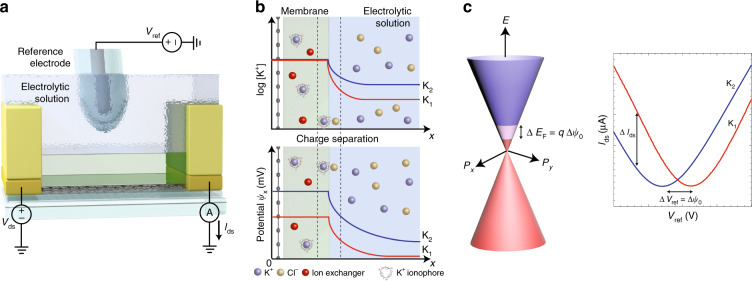
**Graphene ISFET sensing mechanism**. **a** Electrical setup for measuring the current through the graphene channel *I*_ds_ for different electrolytic solutions. *I*_ds_ was monitored at a constant bias *V*_ds_, while the electrolytic potential was varied through the reference electrode *V*_ref_. **b** Mechanism of how changes in analyte concentration affects the ISFET surface potential. The concentration of the target ion is buffered to a constant, and a charge separation layer appears at the membrane/electrolyte interface. A potential difference appears across the interface due to the difference in ion concentration between the membrane and the bulk solution. **c** A change in surface potential leads to a direct shift in the graphene Fermi level and is translated to a shift in the ISFET transfer curve.

The response of each individual ISFET to its respective target ion was measured individually by immersion into electrolytic solutions of controlled concentration, as shown in Fig. 2a. The drain-source currents *I*_ds_ of the graphene ISFETs were measured versus electrolytic gate potential *V*_ref_ regulated through a Ag/AgCl reference electrode at a constant drain-source bias voltage *V*_ds_ = 100 mV.

In the electrolytic solution, the target ion binds to its respective ionophore in the permeable membrane. Fig. 2b illustrates the mechanism by which changes in ion concentration modulate graphene ISFET conduction. As the target ion concentration in the electrolytic solution changes from *a*_1_ to *a*_2_, the concentration in the membrane remains buffered to at a constant value^41,42^. A charge separation layer (diffusion layer) a few nm in thickness appears at the membrane/electrolyte interface. The concentration gradient between the bulk solution and the membrane generates a potential difference across the diffusion layer. Due to the buffered membrane and presence of counter ion exchange sites, the potential across the diffusion layer is *ψ*_0_:

1 $${\psi }_{0}= \, -\alpha \,{\mathrm{ln}}\,10\frac{kT}{zq}\frac{{\mathrm{log}}\,{[a]}_{{\rm{membrane}}}}{{\mathrm{log}}\,{[a]}_{{\rm{solution}}}}\\ = \, {\psi }_{0}^{0}+\alpha\, {\mathrm{ln}}\,10\frac{kT}{zq}{\mathrm{log}}\,{[a]}_{{\rm{solution}}}$$

where ${\psi }_{0}^{0}$ is a surface potential constant, *α* is a dimensionless sensitivity factor between 0 and 1, *k* is Boltzmann’s constant, *T* is temperature, *q* is electric charge, *z* is the valency and [*a*] is the activity of the primary target ion, equivalent to concentration for the range of concentrations explored in this study. As the concentration of the target ion changes from *a*_1_ to *a*_2_, the potential *ψ*_0_ also changes

2 $$\Delta {\psi }_{0}=\alpha\, {\mathrm{ln}}\,10\frac{kT}{zq}(\Delta {\mathrm{log}}\,[a])$$

As a result, Δ*ψ*_0_ is limited thermodynamically and at *T* = 300 K, for an ideal ion sensor with a sensitivity factor *α* = 1, for a target ion with valency *z* = 1, Δ*ψ*_0_ is 59.2 mV per decade change in ion concentration, known as the Nernstian limit.

In the presence of multiple (interfering) ions, we apply the Nikolskii–Eisenman equation, which is a generalization of the Nernst equation (Eq. (1)), to include the contribution to potential from multiple ionic species^9,10^. We use the index *i* to identify physical quantities associated with the primary target ion, and the index *j* to identify physical quantities associated with interfering ions. Thus, *a*_*i*_ is the activity of the primary ion, *a*_*j*_ is the activity of an interfering ion, and *z*_*i*_ and *z*_*j*_ are the valency of the primary and interfering ions, respectively. The potential *ψ*_0*i*_ developed at a membrane for target ion *i* in the Nikolskii–Eisenman theory is,

3 $${{\psi }_{0}}_{i}={{\psi }_{0}^{0}}_{i}+\alpha\, {\mathrm{ln}}\,10\frac{{k}_{B}T}{{z}_{i}q}{\mathrm{log}}\,\left({a}_{i}+\sum_{j\ne i}{K}_{ij}{a}_{j}^{{z}_{i}/{z}_{j}}\right)$$

where *K*_*ij*_ is the selectivity coefficient of the *i*-selective sensor towards interfering ion *j*. For a set of *N* ions, a suite of ISFETs targeting each ion 1 ≤ *i* ≤ *N* can be characterized such that the selectivity coefficients *K*_*ij*_ are known for all 1 ≤ *i* ≤ *N* and 1 ≤ *j* ≤ *N*, with *K*_*ii*_ = 1 by definition. A measurement of *N* potentials *ψ*_0*i*_ from *N* ISFETs will thus allow accurate determination of *N* ion concentrations *a*_*i*_, assuming *K*_*ij*_, ${\psi }_{0i}^{0}$ and *z*_*i*_ have been determined. Importantly, the determination of the *a*_*i*_ from the measured *ψ*_0*i*_ requires the simultaneous solution of *N* nonlinear equations. Additionally, it is important to select the *N* ions carefully such that no ionic species which may cause non-negligible interference is excluded from measurement. Therein lies an advantage of graphene ISFETs, where it is comparatively simple to fabricate multiple sensors as compared to other potentiometric devices.

For a graphene ISFET, the graphene Fermi level is correlated with *ψ*_0_ directly

4 $$\Delta {E}_{{\rm{F}}}=q\frac{{C}_{{\rm{gate}}}}{{C}_{{\rm{q}}}+{C}_{{\rm{gate}}}}\Delta {V}_{{\rm{ref}}}+q\Delta {\psi }_{0}$$

where *C*_q_ is the graphene quantum capacitance and *C*_gate_ is the gate capacitance. Since *C*_gate_ is much larger than *C*_q_ in a well-designed graphene ISFET^18,34^, the capacitive voltage division ratio approaches unity. Thus, at a constant reference potential *V*_ref_, the change in surface potential Δ*ψ*_0_ shifts the graphene Fermi level Δ*E*_F_ = *q*Δ*ψ*_0_ as illustrated in Fig. 2c. This translates to a direct shift of the transfer characteristics curve by Δ*ψ*_0_. Consequently, there are two ways to measure the ion concentration with the graphene ISFETs, either by monitoring the reference potential *V*_np_ that must be applied to reach charge neutrality where graphene conductivity is minimum, or by measuring the current *I*_ds_ at a constant reference potential *V*_ref_ and constant bias potential *V*_ds_. Importantly, the limited on/off current ratio in a graphene ISFET does not directly limit ISFET signal to noise ratio in either mode of operation. The graphene ISFET must provide sufficient small signal gain *g*_m_ = ∂*I*_ds_/∂*V*_ref_ ≈ *C*_q_μ*V*_ds_ such that the signal to noise ratio is limited by potential fluctuations at the graphene ISFET input, and not the additive noise of subsequent amplifiers or signal acquisition stages. As has been previously shown, the combination of high capacitance *C*_q_ ≈ 1 μF cm^−1^ and high mobility *μ* ≈ 2000 cm^2^ V^−1^ s^−1^ with modest bias *V*_ds_ = 100 mV gives sufficient gain *g*_m_ ≈ 0.2 mA/V to reach input noise limited performance^18^.

### ISFET response

**Fig. 3:**
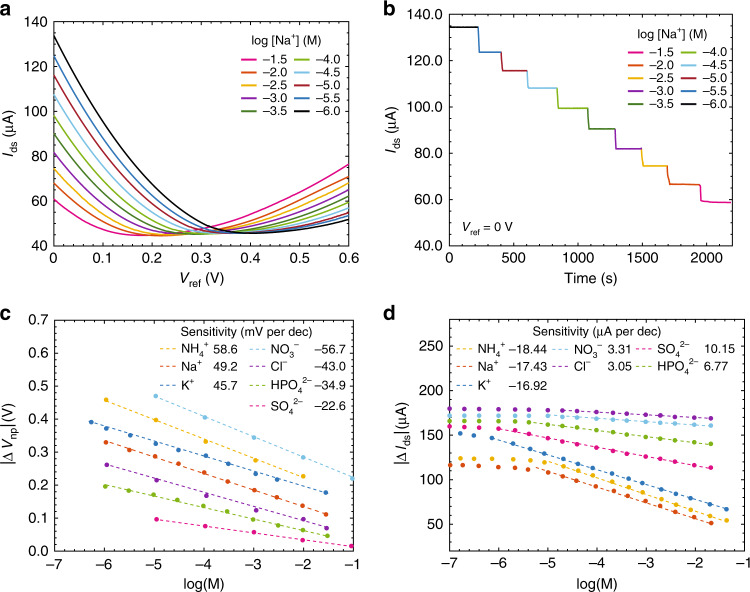
**ISFET sensitivities with respect to their target ions**. **a** The channel current, *I*_ds_, of an Na^+^ ISFET versus *V*_ref_, for different Na^+^ molar concentrations. **b** Continuous real-time measurement of *I*_ds_ for the same Na^+^ ISFET when increasing Na^+^ molar concentrations by half decade steps while keeping *V*_ref_ = 0 V. **c** The changes in *V*_np_ for the different ISFETs with respect to concentrations of their respective target ions, with a linear fit to extract their voltage sensitivities. **d** The changes in *I*_ds_ for the different ISFETs with respect to concentrations of their respective target ions, with a linear fit to extract their current sensitivities.

The ISFETs were first tested independently with respect to their target ion. A representative set of both *V*_np_ and *I*_ds_ measurements for a Na^+^ ISFET is shown in Fig. 3a, b when changing the concentration of NaCl from 10^−6^ to 10^−1.5^ M by half decade steps. The temperature and pH were closely monitored, since both can affect response as will be shown later and were 23 °C and 7.5 pH, respectively. At increasing Na^+^ concentration, the transfer curve shifts uniformly, and we observe a decrease in the potential *V*_np_ required to reach charge neutrality. By measuring *I*_ds_ in real-time at a constant *V*_ref_ when changing the concentration, an instantaneous change in current is observed. The current values at different concentrations match those of Fig. 3a when *V*_ref_ = 0 V. Similar measurements were performed for K^+^, ${{\rm{NH}}}_{4}^{+}$, ${{\rm{NO}}}_{3}^{-}$, ${{\rm{SO}}}_{4}^{2-}$, ${{\rm{HPO}}}_{4}^{2-}$, and Cl^−^ with their respective ISFETs.

A parabolic fit was used to find the potential *V*_np_ of minimum conductance from the measured ISFET transfer curves (Fig. 3a) at every ion concentration, with the resulting dependence on ion concentration plotted in Fig. 3c. A line of best fit was used to calculate ISFET sensitivity. The sensitives between cations and anions differ in sign due to the charge of the target ion. The sensitivity of ISFETs targeting ${{\rm{NH}}}_{4}^{+}$ and ${{\rm{NO}}}_{3}^{-}$ were 58.6 and −56.7 ± 0.2 mV per decade, respectively, approaching the Nernstian limit of 58.8 mV per decade at 23 °C. The sensitivity of ISFETs targeting Na^+^, K^+^, and Cl^−^ were slightly lower at 49.2, 45.7, and −43.0 ± 0.2 mV per decade. The sub-Nernstian response is possibly due to the ionophore to lipophilic salt ratio^42^, which may require further optimization to achieve higher sensitivity. Both ${{\rm{SO}}}_{4}^{2-}$ and ${{\rm{HPO}}}_{4}^{2-}$ are divalent, with a corresponding Nernstian limit of  ~29.4 mV per decade, and measured sensitivities of  −22.6 and  −34.9 ± 0.2 mV per decade, respectively. The apparent super-Nernstian response for the ${{\rm{HPO}}}_{4}^{2-}$ ISFET is due to speciation of phosphate at different pH levels. At a pH of 7.5, 40% of phosphate has the monovalent form ${{\rm{H}}}_{2}{{\rm{PO}}}_{4}^{-}$ ^43^. As a result, a sensitivity falling between the monovalent and divalent Nernstian limits is observed. All ISFETs exhibit linear response down concentrations of 10^−5^ M or lower, which is sufficient for many of the aforementioned applications. Importantly, in all the measurements, a change in counter-ion in the electrolytic solution had no observable effect on the measured sensitivities.

Figure 3d is a plot of the change in steady state graphene ISFET current *I*_ds_ of the different ISFETs versus the molar concentration of their respective target ions, extracted from Fig. 3b and other similar measurements. The current sensitivities of the ISFETs are extracted from linear fits of *I*_ds_ versus ion concentration. The current sensitivity is a function of both the voltage sensitivity and the transistor transconductance. The relatively low current sensitivity for both the ${{\rm{NO}}}_{3}^{-}$ and Cl^−^ ISFETs is due to lower graphene ISFET transconductance, arising from variation in graphene ISFET fabrication. A similar linear response down to concentrations of 10^−5^ M or lower is observed in the current measurements. A typical root mean square noise $\sqrt{\langle {i}_{{\rm{n}}}^{2}\rangle } \,\,{\sim}$ 30 nA in a 60 Hz electrical bandwidth is observed for the ISFETs, resulting in a minimum resolvable concentration of $\sim\! 3\times 1{0}^{-3}\,{\mathrm{log}}\,$ concentration for the cation ISFETs, $\sim\! 3\times 1{0}^{-3}\,{\mathrm{log}}\,$ concentration for the ${{\rm{SO}}}_{4}^{2-}$ ISFET, $\sim\! 5\times 1{0}^{-3}\,{\mathrm{log}}\,$ concentration for the ${{\rm{HPO}}}_{4}^{2-}$, and $\sim\! 2\times 1{0}^{-2}\,{\mathrm{log}}\,$ concentration for both the ${{\rm{NO}}}_{3}^{-}$ and Cl^−^ ISFETs, with a 90% confidence level following IUPAC guidelines^29^. Multiple ISFETs for the same target ion were also fabricated and tested to compare the variability in the fabrication process and performance. It has been found that ionophore membrane induced doping varies amongst graphene ISFETs, but detection limits, sensitivities, and resolution limits are similar. Improved control over the ionophore membrane preparation and drop-casting method is expected to reduce doping variation. Further details are provided in Supplementary Table 2.

**Fig. 4:**
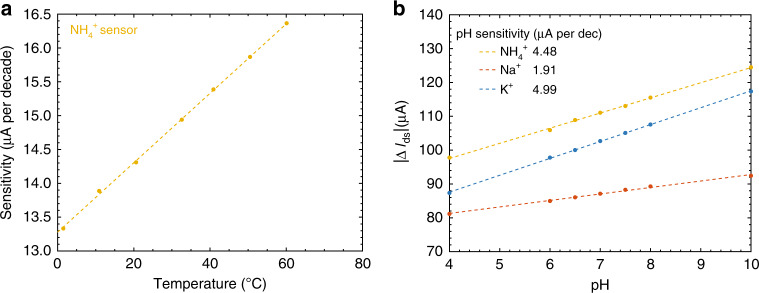
**The effects of temperature and pH on ISFET performance**. **a** The changes in current sensitivity for an ${{\rm{NH}}}_{4}^{+}$ ISFET with respect to temperature. Following the Nernst equation, the sensitivity varies linearly with temperature. **b** The changes in *I*_ds_ for the different cation ISFETs with respect to H^+^ concentrations.

The effects of temperature on graphene ISFET sensitivity was studied by varying the temperature from 1 °C up to 60 °C, and measuring the ISFET current across different concentrations of the target ion under similar bias conditions. A representative measurement is shown for an ${{\rm{NH}}}_{4}^{+}$ ISFET in Fig. 4a. The Nernst Eq. (2) and generalized Nikolskii–Eisenman Eq. (3) both indicate that ISFET sensitivity is expected to vary linearly with absolute temperature *T*, tending to zero sensitivity at 0 K. As shown in Fig. 4a, the measured sensitivity of the ${{\rm{NH}}}_{4}^{+}$ ISFET varies linearly with *T*, with an intercept of *T* = 12 K for zero ion sensitvity inferred from the linear fit, in approximate agreement with theory. The pH of the analyte solution also affects ISFET response, as protons can interfere with the ionophore. The response of ISFETs targeting the cations Na^+^, K^+^, and ${{\rm{NH}}}_{4}^{+}$ to pH was also measured at a constant temperature under similar bias conditions, as shown in Fig. 4b. The ISFETs exhibit a pH sensitivity much smaller than the sensitivity to the target ions as shown in Fig. 3d, and interference from other ionic species are of greater concern as discussed below.

## Discussion

**Fig. 5:**
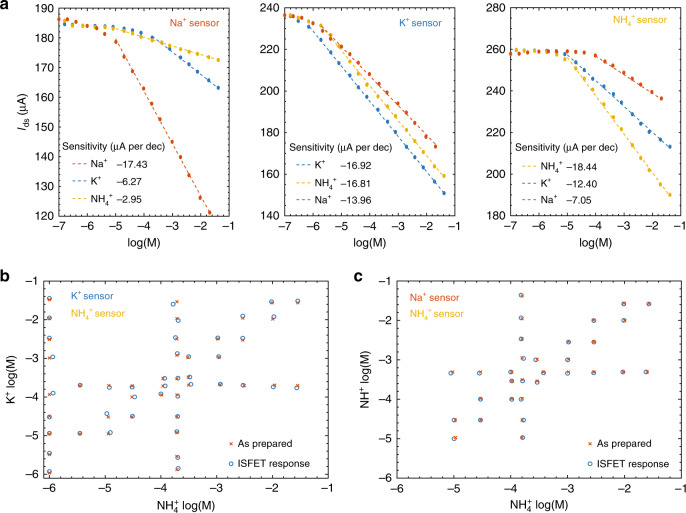
**Overcoming ISFET cross sensitivities**. **a** The changes in *I*_ds_ for the different cation ISFETs with respect to concentrations of the cations, using the separate solution method. Linear fits were used to extract the Nikolskii selectivity coefficients. **b** Comparison of the measured K^+^ and ${{\rm{NH}}}_{4}^{+}$ concentrations by applying the Nikolskii-Eisenman formalism and the as prepared concentrations. The as prepared concentrations are determined from the solution volume and mass of the added salts. **c** Comparison of the measured Na^+^ and ${{\rm{NH}}}_{4}^{+}$ concentrations by applying the Nikolskii-Eisenman formalism and the as prepared concentrations.

The ISFETs’ response to interfering ions was studied by the separate solution method and confirmed with mixed solution method (the fixed interference method)^44,45^. For the separate solution method, the ISFETs were placed in the same solution and the ISFET currents *I*_ds_ were measured concurrently in real-time while varying the ionic concentration. Only one salt was added to de-ionized water to prepare the solution for each experiment. The currents *I*_ds_ were measured with one common reference electrode, where *V*_ref_ = −0.2 V, and a bias *V*_ds_ = 0.1 V was applied to each ISFET. The reference potential *V*_ref_ was chosen to ensure that all ISFETs were operating at a point of high transconductance, far from their respective conductance minima where transconductance vanishes. Figure 5a illustrates the Na^+^, K^+^, and ${{\rm{NH}}}_{4}^{+}$ ISFETs’ response to the different cations used in this study, with linear fits applied to the measured current versus log concentration. The response to the other cations is much higher than with respect to pH from the earlier measurements. Similar measurements were performed for ISFETs sensitive to anions, as shown in Supplementary Fig. 2.

From these measurements, the selectivity coefficients *K*_*ij*_ were extracted and used to generate a series of equations to estimate ion concentration in multiple analyte solutions. Real-time ISFET response is more easily measured in the form of ISFET current *I*_ds_ rather than neutrality point voltage *V*_np_, since the latter requires a sweep of reference voltage *V*_ref_. The Nikolskii–Eisenman Eq. (3) is thus rewritten for current analysis,

5 $${I}_{i}={I}_{i}^{0}+{s}_{ii}\,{\mathrm{log}}\,\left({a}_{i}+\sum_{j\ne i}{K}_{ij}{a}_{j}^{{s}_{ij}/{s}_{ii}}\right)$$

where *I*_*i*_ is the current *I*_ds_ for sensor *i*, ${I}_{i}^{0}$ is the current constant, and *s*_*ii*_ is the current sensitivity for the *i*th sensor for the target ion *i*. Since the ISFETs have different sensitivities to different ions despite having the same valency, the activity *a*_*j*_ of the interfering ions is raised to the power of the *sensitivity* ratio instead of the valency ratio. For an ISFET placed in a solution only containing its respective target ion *i*, Eq. (5) becomes

6 $${I}_{i}={I}_{i}^{0}+{s}_{ii}\,{\mathrm{log}}\,({a}_{i})$$

which is a linear equation between ISFET current *I*_*i*_ and log ion concentration ${\mathrm{log}}\,({a}_{i})$, allowing *s*_*ii*_ to be identified. When the current of ISFET *i* is measured versus the concentration of an interfering ion *j* with all other ion concentrations negligible, Eq. (5) becomes

7 $${I}_{i}={I}_{i}^{0}+{s}_{ii}\, {\mathrm{log}}\,({K}_{ij})+{s}_{ij}{\mathrm{log}}\,({a}_{j})$$

which is also a linear equation between current *I*_*i*_ and log concentration ${\mathrm{log}}\,({a}_{j})$. The sensitivity *s*_*ij*_ can be identified from a linear fit of *I*_*i*_ versus ${\mathrm{log}}\,({a}_{j})$ in Fig. 5a. The constant current ${I}_{ij}^{0}={I}_{i}^{0}+{s}_{ii}\,{\mathrm{log}}\,({K}_{ij})$ allows the Nikolskii selectivity coefficient *K*_*ij*_ to be determined,

8 $${\mathrm{log}}\,{K}_{ij}=\frac{{I}_{ij}^{0}-{I}_{i}^{0}}{{s}_{ii}}$$

Tables 2 and 3 show the Nikolskii coefficients for the cation and anion ISFETs, respectively. For the cations, the K^+^ ISFET is the least selective, giving the greatest response to other cations. For the anions, due to the difference in valency, both SO${}_{4}^{2-}$ and ${{\rm{HPO}}}_{4}^{2-}$ ISFETs are more sensitive to ${{\rm{NO}}}_{3}^{-}$ than their respective primary ions. As for the Cl^−^ ISFET, it is more sensitive to ${{\rm{HPO}}}_{4}^{2-}$ than Cl^−^, an evident limitation of the ionophore membrane. The Nikolskii selectivity coefficients *K*_*ij*_ extracted by the separate solution method were confirmed via the mixed solution fixed interference method. In the fixed interference method, each ISFET’s response was measured against a constant background (BG) activity of an interfering ion *a*_*j*_(BG), while varying the activity of the target ion *a*_*i*_. The Nikolskii selectivity coefficients *K*_*ij*_ are then calculated as ${K}_{ij}={a}_{i}{\rm{(DL)}}/\!{a}_{j}{{\rm{(BG)}}}^{{z}_{i}/{z}_{j}}$, where *a*_*i*_(DL) is the ISFET detection limit for the target ion in the presence of *a*_*j*_(BG)^45,46^. A comparison of *K*_*ij*_ extracted by the two methods reveals that the coefficients agree to within 1% in log activity units (see Supplementary Table 3), signifying that either method can be used for coefficient extraction. Furthermore, the selectivity coefficients are within the same order of magnitude of values reported in the literature for the same Na^+^ ^47^, K^+^ ^48,49^ and ${{\rm{NH}}}_{4}^{+}$ ^50,51^ ionophores, with variation amongst literature results attributed to variation in the ionophore/salt ratio of the ionophore membrane^46^.

**Table 2:** Nikolskii selectivity coefficients *K*_*ij*_ for the three cation ISFETs with respect to three cations.

	Na^+^	K^+^	${\mathrm{NH}}_{4}^{+}$
Na^+^ ISFET	1	9.13 × 10^−4^	1.99 × 10^−4^
K^+^ ISFET	6.45 × 10^−2^	1	3.16 × 10^−1^
${\rm{NH}}_{4}^{+}$ ISFET	2.13 × 10^−3^	2.14 × 10^−2^	1

**Table 3:** Nikolskii selectivity coefficients *K*_*ij*_ for the four anion ISFETs with respect to four anions.

	NO${}_{3}^{-}$	SO${}_{4}^{2-}$	HPO${}_{4}^{2-}$	Cl^−^
${\rm{NO}}_{3}^{-}$ ISFET	1	1.47 × 10^−2^	6.11 × 10^−3^	1.98 × 10^−2^
${\rm{SO}}_{4}^{2-}$ ISFET	8.85 × 10^3^	1	8.27 × 10^−3^	3.75 × 10^−1^
${\rm{HPO}}_{4}^{2-}$ ISFET	1.66 × 10^0^	1.09 × 10^−4^	1	2.10 × 10^−3^
Cl^−^ ISFET	1.51 × 10^−3^	1.38 × 10^−7^	1.28 × 10^2^	1

With offset current ${I}_{i}^{0}$, sensitivity *s*_*ij*_ and selectivity *K*_*ij*_ determined for all ions *i*, *j*, ion concentrations *a*_*i*_ can be estimated from simultaneously measured ISFET currents *I*_*i*_ using Eq. (5) in multi-analyte solutions containing ions *i*, *j*. As will be shown, ion concentration estimation of a multi-ion analyte is accurate in the absence of strongly interfering ions in the analyte that are absent in the ISFET array. Note that numerical methods are required to solve for the ion concentrations *a*_*i*_ from the currents *I*_*i*_ because of the non-linear nature of Eq. (5) in the presence of more than one non-zero concentration *a*_*i*_.

For simplicity, the Nikolskii–Eisenman formalism was first tested with two ISFETs working simultaneously in electrolytic solutions containing two ions of the same charge across a wide range of concentrations. In one case, K^+^ and ${{\rm{NH}}}_{4}^{+}$ concentrations were measured with ISFETs targeting K^+^ and ${{\rm{NH}}}_{4}^{+}$, and in a second case Na^+^ and ${{\rm{NH}}}_{4}^{+}$ concentrations were measured with ISFETs targeting Na^+^ and ${{\rm{NH}}}_{4}^{+}$. These pairs were chosen because of the strong cross-sensitivity of the ISFETs to these ion pairs. The respective ISFETs were placed in the electrolytic solutions, where their currents were measured with reference potential *V*_ref_ = −0.2 V and an independently applied bias *V*_ds_ = 0.1 V. The ion concentrations were calculated from the ISFET currents using the Nikolskii–Eisenman equations Eq. (5), and compared with the as prepared concentrations determined from the volume and mass of the added salts, with the resulting comparison shown in Figs. 5b and c. The concentrations calculated from the ISFET currents are accurate to within $\pm \!0.01\:\,{\mathrm{log}}\,$ concentration units, even when the ratio of ion concentrations exceeds four orders of magnitude.

**Fig. 6:**
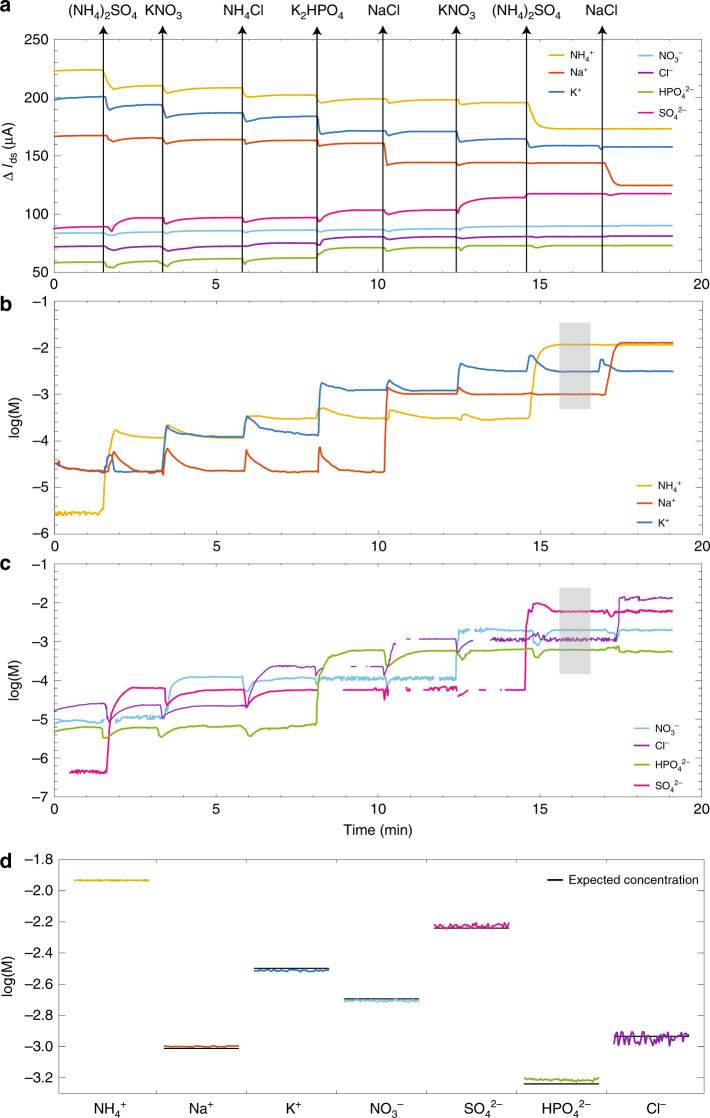
**Real time and simultaneous measurements of ion concentrations in a multi-ion electrolytic solution**. **a** The change in current Δ*I*_ds_ = Δ*I*_*i*_ versus time *t* for all seven ISFETs measured concurrently in a multi-ion electrolytic solution, spiked at different time intervals with salt mixtures as illustrated by the arrows. **b**, **c** The calculated concentrations *a*_*i*_ of the cations and anions, respectively, from the measured currents as determined from the Nikolskii–Eisenman equations. d The accuracy of concentration estimation by the ISFET sensor array is analyzed by comparing the concentrations calculated from the ISFET array currents in the shaded time interval with the concentration determined from the volume and mass of the added salts.

The complete ISFET array performance was then tested with multi-ion electrolytic solutions containing all seven ions. The ISFETs were placed in a solution and their respective currents *I*_ds_ = *I*_*i*_ were measured in real time with reference potential *V*_ref_ = −0.2 V and independently applied bias *V*_ds_ = 0.1 V. The electrolytic solution was spiked with different salt mixtures to change ion concentrations, and the instantaneous changes in the ISFET currents versus time *t* were observed as shown in Fig. 6a.

The ion concentrations of the solution were calculated using the Nikolskii–Eisenman equations Eq. (5) applied to the measured currents *I*_*i*_ at each time *t*. The cations and anions were solved separately since the counter-ions were not found to have any observable effect on sensor response. Figs. 6b and c show the calculated concentrations *a*_*i*_ for cations and anions, respectively, versus time *t* corresponding to the ISFET currents measured in Fig. 6a. Despite the ISFETs responding to interfering ions, the changes in the calculated concentrations only occurred when their respective ions were added. The solution of the nonlinear system of equations failed at some points in time for the anions, where no real solutions can be determined, and these points are omitted from the plot. The failure is most likely due to the comparatively poor current sensitivity of both the Cl^−^ and ${{\rm{NO}}}_{3}^{-}$ ISFETs.

The seven ion concentrations calculated from the seven ISFET currents *I*_*i*_ are compared with the theoretical concentrations determined from the volume and mass of the added salts in Fig. 6d. A one minute time interval represented by the gray shaded area in both Fig. 6b and c was chosen for the comparison. The cation concentrations calculated from ISFET currents are accurate to within $\pm \!0.01\:\,{\mathrm{log}}\,$ concentration units. The anion concentrations are accurate to within $\pm \!0.05\:\,{\mathrm{log}}\,$ concentration units due to lower anion selectivity and lower anion sensitivities. These accuracies are on par with those achieved by spectrophotometric and chromatographic techniques. Importantly, changes in ISFET performance will require recalibration of selectivity coefficients to ensure accurate results.

**Fig. 7:**
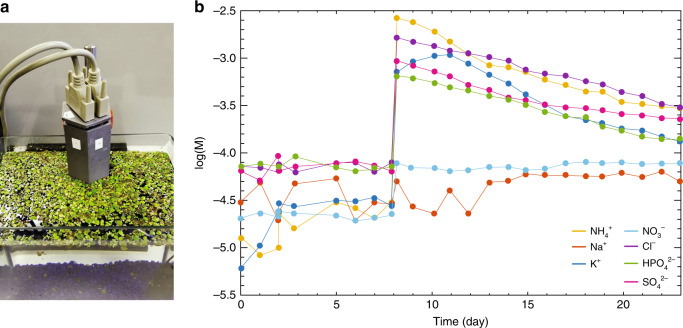
**Measuring ion concentrations in an aquarium**. **a** An optical image of the ISFET array in an aquarium containing duckweed. **b** The measured ion concentrations in the aquarium over time. The concentrations were calculated from the measured currents using the series of Nikolskii–Eisenman equations. After the eighth day, salts were added to the aquarium as nutrients for the plants.

Lastly, the ISFET array was tested in an aquarium environment. The ion concentrations were monitored in an aquarium with *lemnoideae lemna*, commonly known as duckweed, as shown in Fig. 7. *Lemnoideae lemna* is a flowering aquatic plant that is ubiquitous throughout the world. To sustain growth, *lemnoideae lemna* absorbs nutrients including the ions K^+^, ${{\rm{NH}}}_{4}^{+}$, Cl^−^, ${{\rm{HPO}}}_{4}^{2-}$ and ${{\rm{SO}}}_{4}^{2-}$^52,53^. After placing the plants in an aquarium only containing tap water, the ion concentrations were monitored with the ISFET array once a day over a period of three weeks. The plants were provided light 12 hours per day. After one week, (NH_4_)_2_SO_4_, K_2_HPO_4_ and NH_4_Cl were added to the aquarium to set the ion concentrations at  ~3 × 10^−3^ M for ${{\rm{NH}}}_{4}^{+}$ and Cl^−^, and  ~5 × 10^−4^ for K^+^, ${{\rm{SO}}}_{4}^{2-}$ and ${{\rm{HPO}}}_{4}^{2-}$. The ion concentrations *a*_*i*_ versus time *t* as measured by the ISFET array are shown in Fig. 7b, where it can be seen that the K^+^, ${{\rm{NH}}}_{4}^{+}$, Cl^−^, ${{\rm{HPO}}}_{4}^{2-}$, and ${{\rm{SO}}}_{4}^{2-}$ concentrations decrease with time upon introduction to the aquarium. By the end of the third week of observation, the ion concentrations are reduced by 70–80%, as reported in  previous studies of *lemnoideae lemna*^52,53^. To confirm that the decrease in ion concentrations was indeed due to intake of nutrients by the aquatic plants, a control experiment was performed simultaneously, under the same conditions, but in the absence of aquatic plants. The ion concentrations as measured by the ISFET array remained constant after adding the salts to the control sample (see Supplementary Fig. 3). It is thus demonstrated that the graphene ISFET array can be applied to ion concentration measurement in more complex environments, such as that of an aquarium.

In conclusion, we have demonstrated that graphene ISFET arrays can overcome the challenge of poor selectivity in potentiometric sensing. Large-area graphene ISFETs can be fabricated from wafer scale graphene by facile methods. The large active area is critical to achieving a high signal-to-noise-ratio and high resolution sensing. We demonstrate that a modified Nikolskii–Eisenman theory to be applied to estimate the concentration *a*_*i*_ of multiple ions from the ISFET currents *I*_*i*_. ISFET array calibration can be performed simply by the separate solution method, by which selectivity and sensitivity coefficients can be determined. Despite the presence of heavily interfering ions, we have demonstrated ion detection limits down to at least 10^−5^ M concentration. Concentrations in multiple ion electrolytes were accurate to within  ±0.01 and $\pm \!0.05\:\,{\mathrm{log}}\,$ concentration units for cations and anions, respectively, and were resolvable to at least $\sim\! 3\times 1{0}^{-2}\,{\mathrm{log}}\,$ M. We demonstrated the operation of the ISFET array in an aquatic environment, monitoring the uptake of ions in an aquarium by aquatic plants over the course of three weeks. These sensor array characteristics exceed the requirements for many real-time ion monitoring applications. The approach outlined here could be expanded upon, by increasing the number of ISFETs to target a wider range of ions tailored to different problems in environmental sensing.

## Methods

### Device preparation

Graphene ISFETs were fabricated via wafer-scale processing methods, schematically illustrated in Fig. 1. A 100 mm diameter graphene monolayer was grown on poly-crystalline Cu foil catalyst (18 μm) via chemical vapor deposition (CVD) in a cold wall CVD Reactor (Aixtron BM) at Graphenea. The Cu foil was chemically treated and thermally annealed prior to graphene growth at 1000 °C at low pressure. The graphene was wet-transferred via a semi-automated process from the Cu foil to a target wafer using a poly(methyl methacrylate) (PMMA) handle and sacrificial etch of the Cu growth substrate.

The target wafers were 100 mm diameter, 500 μm thick fused silica with a 115 nm layer of parylene C, a hydrophobic polymer that minimizes the unintentional doping of graphene and imparts stability^37^. After transferring the graphene onto the substrate, Ti/Au (20 nm/80 nm) contacts were evaporated onto the wafer to form source and drain contacts with the aid of a shadow mask. The wafer was then diced into individual 1.1 × 1.1 cm^2^ devices, and the devices were then mounted on a printed circuit board (PCB) with two part silver epoxy (EpoTek-H20E) to contact the source and drain of each device (Fig. 1b). The PCBs have a 0.8 × 0.5 cm^2^ opening for the graphene FET to be exposed to the analyte solution.

The ionophore membranes were formed on the graphene FETs by drop-casting 50 μL of a pre-prepared mixture onto the graphene through the PCB opening and left to dry overnight in ambient conditions. The membrane creates a seal between the graphene surface and PCB. Two component epoxy (EpoTek-302) was applied and left to cure overnight to encapsulate the back of transistor and prevent electrical contact with the electrolytic solution.

### Ionophore membrane preparation

We prepared ionophore membrane mixtures for K^+^, Na^+^, ${{\rm{NH}}}_{4}^{+}$, and ${{\rm{SO}}}_{4}^{2-}$, using potassium ionophore III (2-dodecyl-2-methyl-1,3-propanediyl bis[N-[5’-nitro(benzo-15-crown-5)-4’-yl] carbamate]), ammonium ionophore I, sodium ionophore X (4-tert-Butylcalix[4]arene-tetraacetic acid tetraethyl ester), and sulfate ionophore I (1,3-[Bis(3-phenylthioureidomethyl)]benzene) as the ionophore, respectively. To prepare the ionophore mixture, 20–25 mg of the ionophore was mixed with 10 mg of lipophilic salt, 330 mg of poly(vinyl chloride) (PVC) and 660 mg of dioctyl sebacate (DOS) plasticizer. The lipophylic salt was potassium tetrakis (4-cholorophenyl) borate (K-TCPB) for cation sensors, and tridodecylmethylammonium chloride (TDMAC) for the anion sensors. The salt provides exchange sites, thus lowering the electrical resistance of the membrane, reducing anionic interference and consequently improving sensitivity^42,46^. The 1:2 ratio of PVC to plasticizer is a standard matrix for ionophore membranes^46,54^. The mixture was dissolved in 4 mL of tetrahydrofuran (THF) and sonicated overnight. All chemicals were sourced from Sigma Aldrich. Commercially available ionophore membrane cocktails from CleanGrow were used to produce ionophore membranes for Cl^−^, ${{\rm{NO}}}_{3}^{-}$, and ${{\rm{HPO}}}_{4}^{2-}$.

### Solution preparation

KCl, KNO_3_, K_2_HPO_4_, NaCl, Na_2_HPO_4_, NH_4_Cl, and (NH_4_)_2_SO_4_ anhydrous salts with ≥99% purity were used to prepare solutions for studying ISFET sensitivity and selectivity. The concentrations were carefully prepared and diluted with de-ionized water (>10 MΩ), using a combination of microbalance and micropipette. All devices, glassware and components were thoroughly rinsed with de-ionized water prior to, and in between, all measurements to minimize the effects of cross-contamination.

### Raman measurements

To investigate the quality of the transferred graphene, the Raman spectrum was measured using a 532 nm laser, with a spot size less than 1 μm and an incident power  <30 μW with a ×100   magnification lens.

### Hall measurements

To investigate the graphene carrier density and Hall mobility, samples were prepared with a van der Pauw contact geometry. A lock-in amplifier (SRS, SR-830) was used to provide a 1 μA rms 17 Hz AC bias current *I*_B_ and simultaneously monitor the transverse AC Hall voltage *V*_H_ while a normally incident magnetic field **B** was swept between ±800 mT. From the relative orientation of *I*_B_, **B**, and *V*_H_, as well as the sign of ∂*V*_H_/∂**B**, the charge carriers were determined to be holes. The hole density *p* was then extracted using the equation *p* = *I*_B_/(*e*∂*V*_H_/∂**B**) and the Hall mobility *μ*_p_ was determined using *μ*_p_ = 1/(*qnR*_s_), where *R*_s_ is the sheet resistance of the sample inferred from van der Pauw measurements.

### Individual ISFET characterization

The response of each individual ISFET to its respective target ion was measured individually by immersion into electrolytic solutions of controlled concentration, as previously reported for example in ^34^. The drain-source currents *I*_ds_ of the graphene ISFETs were measured via a semiconductor analyzer (Keithley 1500B) versus electrolytic gate potential *V*_ref_ regulated through a Ag/AgCl reference electrode (RE, MF-2078, BASi with 3 M NaCl filling electrolyte) at a constant drain-source bias voltage *V*_ds_ = 100 mV. For voltage sensitivity measurements, the range of *V*_ref_ was controlled to  ±0.8 V to prevent electrolysis of the analyte and to limit the current through the electrolytic gate to no more than 0.5% of the measured channel current *I*_ds_. While for current sensitivity measurements, *V*_ref_ was kept constant and at potentials far from their respective conductance minima to ensure that they were operating at a point of high transconductance.

### ISFET array characterization

In the array configuration, the ISFETs drain-source currents *I*_ds_ were measured simultaneously in real-time using a PalmSens4 potentiostat with one common Ag/AgCl reference electrode, where electrolytic gate potential *V*_ref_ = −0.2 V, and the transistors were all biased at *V*_ds_ = 0.1 V.

For the real-time measurements in Fig. 6, the ISFET array was initially placed in a beaker containing 254 mL of DI water. 0.05 mL of 10^−2^ M NH_4_Cl, 0.15 mL of 10^−2^ M K_2_HPO_4_, 0.25 mL of 10^−2^ M KNO_3_, 0.5 mL of 10^−2^ M NaCl, and 1 mL of 10^−4^ M (NH_4_)_2_SO_4_ were added to result in starting concentrations of $-4.666\,\,{\mathrm{log}}\,[{{\rm{K}}}^{+}]$ M, $-5.548\,\,{\mathrm{log}}\,[{{\rm{NH}}}_{4}^{+}]$ M, $-4.703\,\,{\mathrm{log}}\,[{{\rm{Na}}}^{+}]$ M, $-5.006\,\,{\mathrm{log}}\,[{{\rm{NO}}}_{3}^{-}]$ M, $-6.310\,\,{\mathrm{log}}\,[{{\rm{SO}}}_{4}^{2-}]$ M, $-5.225\,\,{\mathrm{log}}\,[{{\rm{HPO}}}_{4}^{2-}]$ M, and $-4.666\,\,{\mathrm{log}}\,[{{\rm{Cl}}}^{-}]$ M. Once the solution was thoroughly mixed with a stir bar, the currents of the ISFETs were measured in real time and salts were added every few minutes in the following order: 1.5 mL of 10^−2^ M (NH_4_)_2_SO_4_, 3 mL of 10^−2^ M KNO_3_, 0.05 mL of 1 M NH_4_Cl, 0.15 mL of 1 M K_2_HPO_4_, 0.25 mL of 1 M NaCl, 0.5 mL of 1 M KNO_3_, 1.5 mL of 1 M (NH_4_)_2_SO_4_ and 3 mL of 1 M NaCl. After each mixture was added, the solution was mixed with a stir bar for a few seconds leading to the small transient in current observed. The ion concentrations were obtained by solving the series of Nikolskii–Eisenman equations (Eq. (5)), where the constants were extracted from the calibration tests.

## Supplementary information

Supplementary Information

## Data Availability

The data that support the findings of this study are available on request from the corresponding authors I.F. and T.S.

## Code availability

The code that supports the findings of this study is available on request from the corresponding authors I.F. and T.S.

## References

[1] Rothberg JM (2011). An integrated semiconductor device enabling non-optical genome sequencing. Nature.

[2] Chapp AD (2018). Measurement of cations, anions, and acetate in serum, urine, cerebrospinal fluid, and tissue by ion chromatography. Physiol. Rep..

[3] *Protocols manual for water quality sampling in Canada* (Canadian Council of Ministers of the Environment, 2011).

[4] *Developing drinking water quaility regulations and Standard*s (World Health Organization, 2018).

[5] Nielsen, S. S. *Food Analysis Laboratory Manual* (Springer International Publishing, 2017).

[6] Gros N, Camòes MF, Oliveira C, Silva MCR (2008). Ionic composition of seawaters and derived saline solutions determined by ion chromatography and its relation to other water quality parameters. J. Chromatogr. A.

[7] Aßmann S, Frank C, Kortzinger A (2011). Spectrophotometric high-precision seawater pH determination for use in underway measuring systems. Ocean Sci..

[8] Bergveld P (2003). Thirty years of ISFETOLOGY what happened in the past 30 years and what may happen in the next 30 years. Sens. Actuators, B.

[9] Nikolskii BP (1937). Theory of the glass electrode. I. theoretical. J. Phys. Chem..

[10] Eisenman G, Rudin DO, Casby JU (1957). Glass electrode for measuring sodium ion. Science.

[11] Bobacka J, Ivaska A, Lewenstam A (2008). Potentiometric ion sensors. Chem. Rev..

[12] Igarashi I, Ito T, Taguchi T, Tabata O, Inagaki H (1990). Multiple ion sensor array. Sens. Actuators B.

[13] Forster RJ, Regan F, Diamond D (1991). Modeling of potentiometric electrode arrays for multicomponent analysis. Anal. Chem..

[14] Forster RJ, Diamond D (1992). Nonlinear calibration of ion-selective electrode arrays for flow injection analysis. Anal. Chem..

[15] Huang X, Yu H, Liu X, Jiang Y, Yan M, Wu D (2015). A dual-mode large-arrayed CMOS ISFET sensor for accurate and high-throughput pH sensing in biomedical diagnosis. IEEE Trans. Biomed. Eng..

[16] Milgrew M, Cumming D (2008). Matching the transconductance characteristics of CMOS ISFET arrays by removing trapped charge. IEEE Trans. Electron Devices.

[17] Moser N, Lande TS, Toumazou C, Georgiou P (2016). ISFETs in CMOS and emergent trends in instrumentation: a review. IEEE Sens. J..

[18] Fakih I, Mahvash F, Siaj M, Szkopek T (2017). Sensitive precise pH measurement with large-area graphene feld-effect transistors at the quantum-capacitance limit. Phys. Rev. Appl..

[19] Knopfmacher O, Tarasov A, Fu W, Wipf M, Niesen B, Calame M, Schönenberger C (2010). Nernst limit in dual-gated si-nanowire fet sensors. Nano Lett..

[20] Nishiguchi K, Clement N, Yamaguchi T, Fujiwara A (2009). Si nanowire ion sensitive field-effect transistors with a shared floating gate. Appl. Phys. Lett..

[21] Lee JS (2011). Silicon nanowire ion sensitive field effect transistor with integrated Ag/AgCl electrode: pH sensing and noise characteristics. Analyst.

[22] Chang S-R, Chen H (2009). A CMOS-Compatible, Low-Noise ISFET Based on High Efficiency Ion-Modulated Lateral-Bipolar Conduction. Sensors.

[23] Georgakilas A (2007). AlGaN/GaN high electron mobility transistor sensor sensitive to ammonium ions. Phys. Stat. Sol..

[24] Parish G (2013). Nitrate ion detection using AlGaN/GaN heterostructure-based devices without a reference electrode. Sens. Actuators B.

[25] Hu W (2019). High-stability pH sensing with a few-layer MoS_2_ field-effect transistor. Nanotechnology.

[26] Banerjee K (2014). MoS_2_ field-effect transistor for next generation label-free biosensors. ACS Nano..

[27] Yeh JA (2011). Highly sensitive pH sensing using an indium nitride ion-sensitive field-effect transistor. Sens. Actuators B.

[28] Cid CC, Riu J, Maroto A, Rius FX (2008). Ion-sensitive field effect transistors using carbon nanotubes as the transducing layer. Analyst.

[29] Inczedy, J., Lengyel, T. & Ure, A. *Compendium of Analytical Nomenclature: Definitive Rules* (International Union of Pure and Applied Chemistry, 1997).

[30] Wang X, Tabakman SM, Dai H (2008). Atomic layer deposition of metal oxides on pristine and functionalized graphene. J. Am. Chem. Soc..

[31] Fu W (2011). Graphene transistors are insensitive to pH changes in solution. Nano Lett..

[32] Fu W (2013). High mobility graphene ion-sensitive field-effect transistors by noncovalent functionalization. Nanoscale.

[33] Koley G (2017). Graphene field effect transistors for highly sensitive and selective detection of K^+^ ions. Sens. Actuators B.

[34] Fakih I (2019). High resolution potassium sensing with large-area graphene field-effect transistors. Sens. Actuators B.

[35] Jørgensen, S., Löffler, H., Rast, W. & Straskraba, M. *Lake and Reservoir Management* (Elsevier, 2005).

[36] Jørgensen, S. & Fath, B. D. *Encyclopedia of Ecology* (Elsevier, 2008).

[37] Sabri, S. S. et al. Graphene field effect transistors with parylene gate dielectric. *Appl. Phys. Lett.* **95**, 242104 (2009).

[38] Ferrari AC, Basko DM (2013). Raman spectroscopy as a versatile tool for studying the properties of graphene. Nat. Nanotechnol..

[39] Rao R, Tishler D, Katoch J, Ishigami M (2011). Multiphonon Raman scattering in graphene. Phys. Rev. B.

[40] Huang PY (2011). Grains and grain boundaries in single-layer graphene atomic patchwork quilts. Nature.

[41] van Hal R, Eijkel J, Bergveld P (1995). A novel description of ISFET sensitivity with the buffer capacity and double-layer capacitance as key parameters. Sens. Actuators, B.

[42] Telting-Diaz M, Bakker E (2001). Effect of lipophilic ion-exchanger leaching on the detection limit of carrier-based ion-selective electrodes. Anal. Chem..

[43] Powell KJ (2005). Chemical speciation of environmentally significant heavy metals with inorganic ligands. Part 1: The Hg^2+^, Cl^−^, OH^−^, CO$${}_{3}^{2-}$$, SO$${}_{3}^{2-}$$, and PO$${}_{3}^{2-}$$ in aquatic systems. Pure Appl. Chem..

[44] Guilbault GG (1976). Recommendations for nomenclature of ion-selective electrodes. Pure Appl. Chem..

[45] Umezawa Y, Bühlmann P, Umezawa K, Tohda K, Amemiya S (2000). Potentiometric selectivity coefficients of ion-selective eletrodes. Pure Appl. Chem..

[46] Bakker E, Bühlmann P, Pretsch E (1997). Carrier-based ion-selective electrodes and bulk optodes. 1. general characteristics. Chem. Rev..

[47] Diamond D (1992). All-solid-state sodium-selective electrode based on a calixarene ionophore in a poly (vinyl chloride) membrane with a polypyrrole solid contact. Anal. Chem..

[48] Kukorelli T (1985). In vivo measurements with a potassium ion-selective microelectrode based on a new bis (crown ether). Analytica Chim. Acta.

[49] Töke L (1990). Novel bis (crown ether)-based potassium sensor for biological applications. Microchimica Acta.

[50] Launay J (2017). Multimodal probe based on isfet electrochemical microsensors for in-situ monitoring of soil nutrients in agriculture. Multidiscip. Digital Publ. Inst. Proc..

[51] Ohba S (2000). Design and synthesis of a more highly selective ammonium ionophore than nonactin and its application as an ion-sensing component for an ion-selective electrode. Anal. Chem..

[52] *Duckweed: A tiny aquatic plant with enormous potential for agriculture and environment* (World Watch, 1997).

[53] Porath D, Pollock J (1982). Ammonia stripping by duckweed and its feasibility in circulating aquaculture. Aquat. Bot..

[54] Gibbons WS, Patel HM, Kusy RP (1997). Effects of plasticizers on the mechanical properties of poly(vinyl chloride) membranes for electrodes and biosensors. Polymer.

