# Supplementary material for: Selective ion sensing with high resolution large area graphene field effect transistor arrays
Source: Nat Commun. 2020 Jun 26;11:3226. doi: 10.1038/s41467-020-16979-y (PMC7320191; doi:10.1038/s41467-020-16979-y)
Supplement: Supplementary file 1 — Supplementary Information [file 41467_2020_16979_MOESM1_ESM.pdf]

Supplemental Information for

Selective ion sensing with high resolution large area graphene field effect transistor arrays

Fakih et al.

Supplementary Table 1

Hall measurements of graphene devices from the same wafer were conducted to study the quality and uniformity of the graphene. Similar to our ISFETs, a 100 mm diameter graphene monolayer grown via CVD was transferred on to a target wafer of 500 μm thick silicon wafer with 300 nm of dry thermal oxide and a 115 nm layer of parylene C. The Si wafer was used for these measurements instead of the fused silica used for the ISFETs in order to conduct back-gated measurements as well.

The graphene devices were fabricated into different areas of van der Pauw contact geometry, where voltage was measured across the contacts for a fixed bias current while sweeping a magnetic field between $\pm$ 800 mT.

Table 1: Comparison of hall mobility and carrier density of different graphene devices from the same wafer.

Active area (m^2)	Hall mobility ($cm^2V^{-1}s^{-1}$)	Carrier density (cm^{-2})
2.5×10^{-11}	1660	8.41×10^{12}
1.6×10^{-9}	2050	8.34×10^{12}
6.4×10^{-9}	1810	9.19×10^{12}
2.6×10^{-8}	1480	6.75×10^{12}
1.0×10^{-7}	1320	7.44×10^{12}
1.6×10^{-6}	1930	8.93×10^{12}
6.6×10^{-6}	2010	8.00×10^{12}
6.6×10^{-6}	1580	9.64×10^{12}
2.6×10^{-5}	1610	7.73×10^{12}

Supplementary Figure 1

The back-gated graphene transfer curve was measured before and after drop-casting the ionophore to study the effect of the ionophore membrane on the graphene. The graphene becomes more n-doped after adding the ionophore with anion membranes being more significant. However, the mobility of graphene is not degraded, but on the contrary, it slightly improves, signifying that the ionophore membrane non-covalently functionalizes the graphene.

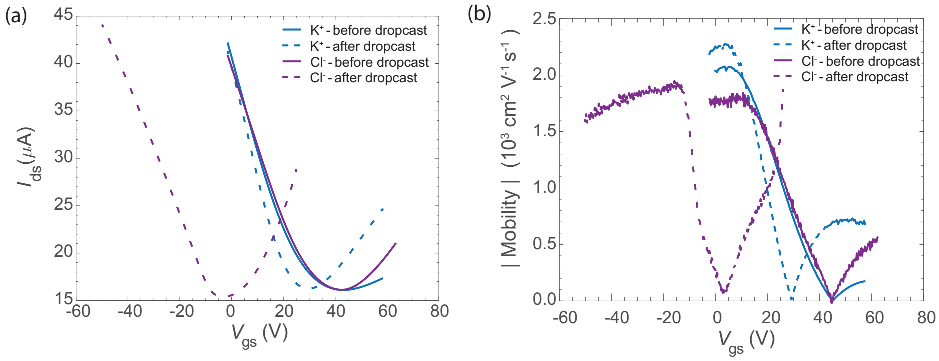

Figure 1: (a) The back-gated graphene transfer characteristic curves before and after drop-casting both cation and anion ionophore membranes. (b) The change in absolute value of field effect mobility of the same graphene ISFETs before and after drop-casting the ionophores.

Supplementary Table 2

Multiple ISFETs for the same target ionophore were fabricated from the same wafer and using the same ionophore membranes. Their performance is compared below. The resolution is calculated to a 90% accuracy level according to IUPAC guidelines[45]. The neutrality point of each sensor at 10^{-3} M for their respective target ion shows the effects of the ionophore membrane on the graphene doping. The variation between the doping between the ISFETs is most likely due to the final few steps of the fabrication process which were done individually: the drop-casting the ionophore, and the application of the encapsulating epoxy. A more automated process will most likely improve uniformity. In terms of detection limit, sensitivity and resolution, there is little difference between the ISFETs for the same target ion. Regardless of the uniformity between the sensors, each sensor must be properly calibrated to ensure accurate measurements in the array.

Table 2: Comparison of ISFET performance for different target ions.

Device	Target Ion	V_{np} at 10^{-3} M (mV)	Detection Limit (M)	Voltage Sensitivity (mV per dec)	Current Sensitivity (µA per dec)	Resolution (log [a] M)
F19146-25	NH_4^+	520	10^{-6}	58.6	18.44	2.8 $\times 10^{-3}$
F19146-26	NH_4^+	430	$10^{-5.5}$	48	15.4	5.6 $\times 10^{-3}$
F19146-31	NH_4^+	106	10^{-6}	56	9.1	
F19146-32	NH_4^+	80	10^{-5}	57	5.4	
F19146-11	K^+	381	10^{-6}	41.3	20.63	3.0×10^{-3}
F19146-12	K^+	472	10^{-8}	38.9	22.22	2.7×10^{-3}
F19146-13	K^+	536	10^{-8}	45.2	18.47	2.6 $\times 10^{-3}$
F19146-33	K^+	435	10^{-5}	45.7	16.92	3.1×10^{-3}
F19146-5	Na^+	245	10^{-5}	48.7	14.42	4.2×10^{-3}
F19146-18	Na^+	261	10^{-5}	49.0	21.79	2.8×10^{-3}
F19146-23	Na^+	267	10^{-5}	49.2	17.43	2.9 $\times 10^{-3}$
F19146-2	HPO_4^{2-}	287	$10^{-4.5}$	27.1	5.47	1.2×10^{-2}
F19146-3	HPO_4^{2-}	318	$10^{-4.5}$	30.0	5.69	7.8×10^{-3}
F19146-24	HPO_4^{2-}	262	10^{-6}	34.9	6.77	5.1 $\times 10^{-3}$

Supplementary Table 3

In order to solve the series Nikolskii-Eisenman equations and calculate the ion concentrations in multi-analyte solutions, the Nikolskii selectivity coefficients were solved for using both the separate solution and mixed solution methods (fixed interference method) and compared. According to IUPAC, both techniques can be used interchangeably, and our results confirm this.

Table 3: Nikolskii selectivity coefficients for the cation ISFETs with respect to the different cations, using the separate solution method and mixed solution method (fixed interference method)

	**Separate Solution Method**			**Mixed Solution Method**		
	$\mathbf{Na^+}$	$\mathbf{K^+}$	$\mathbf{NH_4^+}$	$\mathbf{Na^+}$	$\mathbf{K^+}$	$\mathbf{NH_4^+}$
**Na^+ ISFET**	1	1.18×10^{-3}	2.12×10^{-4}	1	1.07×10^{-3}	2.46×10^{-4}
**K^+ ISFET**	3.46×10^{-2}	1	1.16×10^{-1}	3.32×10^{-2}	1	1.05×10^{-1}
**NH_4^+ ISFET**	2.26×10^{-3}	3.87×10^{-2}	1	2.09×10^{-3}	3.98×10^{-2}	1

Supplementary Figure 2

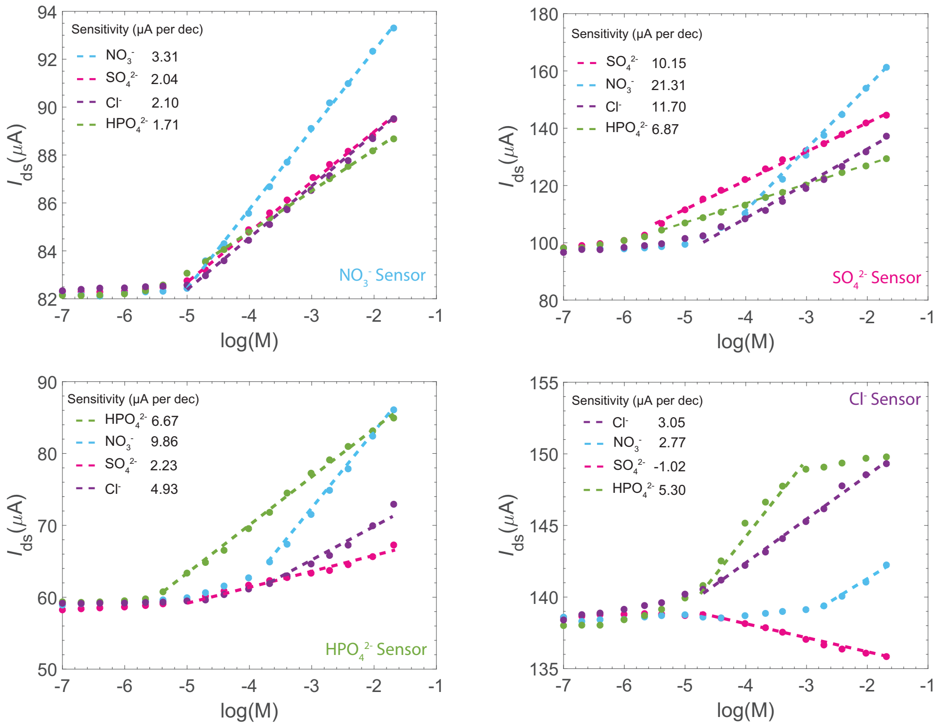

Figure 2: The changes in I_{ds} for the different anion ISFETs with respect to concentrations of the anions, using the separate solution methods. Linear fits were used to extract the Nikolskii selectivity coefficients.

Supplementary Figure 3

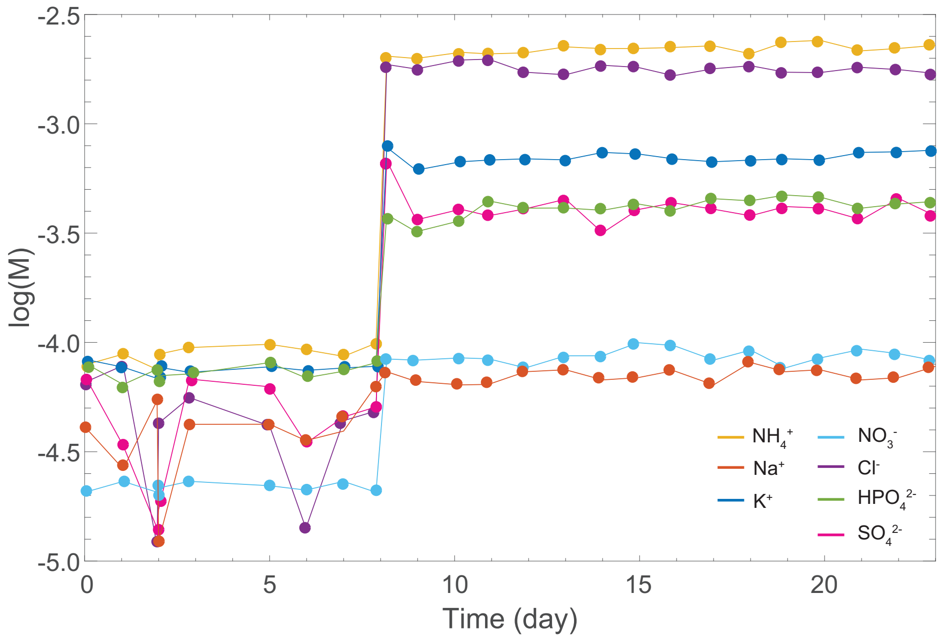

Figure 3: The ion concentration in a control beaker that underwent the same conditions as the aquarium but did not contain any duckweed. The concentrations were calculated from the measured currents using the series of Nikolskii-Eisenman equations.
